# Effect of a brief alcohol counselling intervention on HIV viral suppression and alcohol use among persons with HIV and unhealthy alcohol use in Uganda and Kenya: a randomized controlled trial

**DOI:** 10.1002/jia2.26187

**Published:** 2023-12-06

**Authors:** Sarah B. Puryear, Florence Mwangwa, Fred Opel, Gabriel Chamie, Laura B. Balzer, Jane Kabami, James Ayieko, Asiphas Owaraganise, Elijah Kakande, George Agengo, Elizabeth Bukusi, Stella Kabageni, Daniel Omoding, Melanie Bacon, John Schrom, Sarah Woolf‐King, Maya L. Petersen, Diane V. Havlir, Moses Kamya, Judith A. Hahn

**Affiliations:** ^1^ Division of HIV, ID and Global Medicine University of California San Francisco California USA; ^2^ Infectious Diseases Research Collaboration Mbarara Uganda; ^3^ Kenya Medical Research Institute Kisumu Kenya; ^4^ Division of Biostatistics School of Public Health University of California Berkeley Berkeley California USA; ^5^ National Institute of Allergy and Infectious Diseases Bethesda Maryland USA; ^6^ Department of Psychology Syracuse University Syracuse New York USA; ^7^ Department of Medicine Makerere University College of Health Sciences Kampala Uganda

**Keywords:** HIV, viral suppression, alcohol use, brief counselling intervention, sub‐Saharan Africa, randomized controlled trial

## Abstract

**Introduction:**

Unhealthy alcohol use significantly contributes to viral non‐suppression among persons with HIV (PWH). It is unknown whether brief behavioural interventions to reduce alcohol use can improve viral suppression among PWH with unhealthy alcohol use in sub‐Saharan Africa (SSA).

**Methods:**

As part of the SEARCH study (NCT04810650), we conducted an individually randomized trial in Kenya and Uganda of a brief, skills‐based alcohol intervention among PWH with self‐reported unhealthy alcohol use (Alcohol Use Disorders Identification Test–Consumption [AUDIT‐C], prior 3 months, ≥3/female; ≥4/male) and at risk of viral non‐suppression, defined as either recent HIV viral non‐suppression (≥400 copies/ml), missed visits, out of care or new diagnosis. The intervention included baseline and 3‐month in‐person counselling sessions with interim booster phone calls every 3 weeks. The primary outcome was HIV viral suppression (<400 copies/ml) at 24 weeks, and the secondary outcome was unhealthy alcohol use, defined by AUDIT‐C or phosphatidylethanol (PEth), an alcohol biomarker, ≥50 ng/ml at 24 weeks.

**Results:**

Between April and September 2021, 401 persons (198 intervention, 203 control) were enrolled from HIV clinics in Uganda (58%) and Kenya (27%) and alcohol‐serving venues in Kenya (15%). At baseline, 60% were virally suppressed. Viral suppression did not differ between arms at 24 weeks: suppression was 83% in intervention and 82% in control arms (RR: 1.01, 95% CI: 0.93–1.1). Among PWH with baseline viral non‐suppression, 24‐week suppression was 73% in intervention and 64% in control arms (RR 1.15, 95% CI: 0.93–1.43). Unhealthy alcohol use declined from 98% at baseline to 73% in intervention and 84% in control arms at 24 weeks (RR: 0.86, 95% CI: 0.79–0.94). Effects on unhealthy alcohol use were stronger among women (RR 0.70, 95% CI: 0.56–0.88) than men (RR 0.93, 95% CI: 0.85–1.01) and among participants with a baseline PEth⩽200 ng/ml (RR 0.68, 95% CI: 0.53–0.87) versus >200 ng/ml (RR 0.97, 95% CI: 0.92–1.02).

**Conclusions:**

In a randomized trial of 401 PWH with unhealthy alcohol use and risk for viral non‐suppression, a brief alcohol intervention reduced unhealthy alcohol use but did not affect viral suppression at 24 weeks. Brief alcohol interventions have the potential to improve the health of PWH in SSA by reducing alcohol use, a significant driver of HIV‐associated co‐morbidities.

## 1 INTRODUCTION

Unhealthy alcohol use is common among persons with HIV (PWH) [1]. Sub‐Saharan Africa (SSA) is home to 68% of the 34 million PWH worldwide [2] and has the highest global prevalence of heavy episodic drinking [3]. Unhealthy alcohol use, defined as drinking above recommended limits [4], has been associated with lower antiretroviral therapy (ART) adherence [5, 6], lower HIV viral suppression [7, 8, 9] and increased sexual risk behaviours [10, 11], contributing to onward HIV transmission. Scalable interventions to reduce alcohol use and improve viral suppression among PWH in SSA are critically needed.

Health services addressing alcohol consumption are limited in SSA [12, 13]. Only a few studies of scalable, brief behavioural interventions for alcohol use reduction specific to PWH in SSA have been conducted [14, 15, 16, 17, 18, 19]. A global meta‐analysis of alcohol counselling interventions for PWH found small but significant reductions in alcohol consumption and increases in HIV viral suppression [20], but a subsequent systematic review did not find effects on either [21]. It remains unknown whether brief behavioural interventions can impact viral suppression and alcohol use among PWH in SSA.

We conducted a randomized controlled trial (RCT) to compare the effectiveness of a brief, culturally adapted [17, 22] skills‐based alcohol counselling intervention with standard care for PWH with unhealthy alcohol use and risk for viral non‐suppression. Our primary outcome was viral suppression at 24 weeks after enrolment. Our secondary outcome was changes in unhealthy alcohol use at 24 weeks, assessed by the Alcohol Use Disorders Identification Test‐Consumption (AUDIT‐C) and phosphatidylethanol (PEth), an alcohol biomarker. We hypothesized that the intervention would increase viral suppression and reduce unhealthy alcohol use compared to standard care.

## 2 METHODS

### 2.1 Trial design

During the pilot phase (Phase A) of the SEARCH‐SAPPHIRE trial (NCT04810650), we conducted a two‐arm, individual RCT of the impact of a brief, culturally adapted skills‐based alcohol counselling intervention on HIV viral suppression among PWH with heavy alcohol use and at risk for viral non‐suppression.

### 2.2 Study setting

Between April and September 2021, we screened participants at rural government‐run health centres in three communities in southwestern Uganda and five communities in Western Kenya and at alcohol‐serving venues in the Kenyan study communities. In Uganda, COVID‐19 restrictions closed all bars and nightclubs from March 2020 until January 2022, precluded recruitment at these sites.

### 2.3 Recruitment

For clinic‐based recruitment, the study team performed chart and registry reviews and met with clinic staff to pre‐identify adults potentially eligible for the study. Venue‐based recruitment used methods developed previously [23]. Briefly, study staff met with community representatives to identify alcohol‐serving venues, and distributed recruitment cards at venues that invited patrons and staff to local government‐run clinics for free screening for hypertension, diabetes, malaria and HIV, with reimbursement for transportation paid at screening (500KSH, approximately US$5). Adults not known to have HIV underwent testing per country guidelines. We linked adults who tested positive to HIV care and invited all PWH (new or prior diagnosis) to study screening.

### 2.4 Participants

The study inclusion criteria were ≥18 years of age, AUDIT‐C (modified to prior 3 months) [24] positive for unhealthy alcohol use (≥4 for men, ≥3 for women) [25], HIV positive and one of the following risk factors for non‐suppression: HIV RNA non‐suppression (>400 copies/ml) in the prior 12 months, missed clinic visit (>2 weeks to ≤90 days from the last scheduled visit) in the past 6 months, out of care (>90 days from last scheduled visit) in the past 6 months or new HIV diagnosis (not yet on ART or ART start <4 weeks). Exclusion criteria were plans to move out of the study area within 6 months, enrolment in other SEARCH SAPPHIRE trials or inability to give informed consent. Given the shortage of mental health professionals in Africa [26], persons with a screening AUDIT‐C score of ≥8, indicative of high risk for severe alcohol use disorder, were enrolled and additionally offered referral to their physician for further care outside of the intervention.

### 2.5 Enrolment and randomization

We randomized participants 1:1 to the alcohol counselling intervention or standard‐of‐care (control) using stratified block randomization (block size = 2 and 4), computer‐generated and stratified by country. Participants were not masked to the randomization arm, but the study statistician (LBB) was masked until trial completion.

### 2.6 Study procedures

At baseline, we administered a structured questionnaire which included demographics, AUDIT‐C adapted to the prior 3 months, ART use, and current tobacco and other substance use assessments. We completed baseline study visits within 14 days of enrolment. After 24 weeks from baseline, study staff administered the AUDIT‐C for the prior 3 months.

Laboratory assessments included HIV‐1 RNA viral load, CD4 cell count and PEth level, a quantitative biomarker of recent alcohol consumption, at baseline, and viral load and PEth at week 24. We prepared dried blood spots onto Whatman 903 cards, and shipped them in batches to a commercial laboratory, the United States Drug Testing Laboratory (USDTL, Des Plaines, IL), for quantification of PEth (16:0/18:1 homologue), with an 8 ng/ml lower limit of quantification [27]. PEth correlates with the total volume of alcohol consumed over the past 2–4 weeks and forms only in the presence of alcohol, thus is highly specific [28]. PEth ≥50 ng/ml is consistent with unhealthy drinking [29] and PEth>200 ng/ml is consistent with chronic excessive drinking [30].

Participants received a transport reimbursement (500KSH or 20,000UGX, approximately US$5 in 2021) for in‐person study visits at baseline, week 12 (intervention only) and week 24.

### 2.7 Intervention

The intervention was a multi‐session brief alcohol counselling intervention based on the Information, Motivation, and Behavioral skills (IMB) model [31] and implemented in a prior intervention shown to be efficacious for reducing alcohol use among women in the United States [32]. The intervention was adapted for East Africa using iterative cultural adaptation to modify key intervention characteristics for cultural relevance and saliency, as previously described [22].

The intervention included two in‐person counselling sessions, performed at baseline and week 12, with brief “booster” phone‐based counselling session performed every 3 weeks between in‐person sessions. The in‐person sessions were workbook‐guided and designed to reflect on the consequences of alcohol use on one's health and HIV treatment, identify risky moods and situations that precede alcohol use, build skills for alternative behaviours and set individualized goals to decrease alcohol intake. Booster calls were tailored to participants’ goals and designed to check progress and provide positive reinforcement and encouragement as appropriate. Intervention participants received viral load and adherence counselling based on results from the baseline study visit by study staff at the 3‐week phone call and by clinic staff at their post‐baseline regular clinic visit.

Counselling was conducted by lay counsellors trained by a licensed clinical psychologist (SWK) on the basic Motivational Interviewing skills and implementation of the manualized intervention. SWK provided ongoing group supervision for the counsellors.

### 2.8 Control arm

Participants randomized to the control arm received brief advice on the harmful effects of alcohol and safe levels of drinking at the baseline visit in line with the country standard of care, viral load feedback and adherence counselling at their post‐baseline regular clinic visit, and an invitation to receive the intervention counselling and materials after 48 weeks.

Participants in both study arms had access to a toll‐free hotline to reach study staff with questions or problems during the study.

### 2.9 Fidelity

Trained qualitative researchers assessed intervention fidelity using an adapted checklist to rate counsellors’ content inclusion and counselling skills during observation of randomly selected in‐person sessions (*n* = 5/counsellor) [17, 33]. Performance was discussed in group supervision sessions.

### 2.10 Outcomes

The primary outcome was viral suppression at 24 weeks defined by HIV‐1 RNA<400 copies/ml, based on available assays in country and to avoid misclassification of low‐level viral blips as non‐suppression. A 24‐week endpoint was selected to align with participants’ standard HIV care visits to minimize inconvenience, travel time and cost to participants, as well as a burden on clinic study sites and to allow time for effect of the 12‐week counselling visit. In primary analyses, participants without an endpoint viral load were assumed to be disengaged from care and unsuppressed. Individuals who moved out of the study region, died or withdrew were excluded. A secondary outcome was unhealthy alcohol use, defined as a composite of AUDIT‐C positive (score ≥3 for women, ≥4 for men) or PEth ≥50 ng/ml at 24 weeks. This composite was used to increase the overall sensitivity by combining two measures with high specificity.

### 2.11 Statistical analyses

Based on a two‐sample test for proportions, a sample size of 200 participants/arm would provide 80% power to detect a 14% absolute increase [34] in viral suppression at 24 weeks from 40% in the control group.

Primary and secondary outcomes were compared by trial arms using targeted minimum loss‐based estimation to adaptively adjust for baseline predictors for improved efficiency while preserving nominal type‐I error control under the null [35, 36]. Baseline predictors were specified *a priori* and included sex, age, country, baseline HIV‐1 RNA >400 copies/ml, baseline PEth (log10 transformed) and baseline AUDIT‐C. Effect estimates were on the relative scale; thus, we reported as risk ratios (RR) and corresponding 95% confidence intervals to test the null hypothesis that the intervention did not improve outcomes. To explore the heterogeneity of intervention effects, we repeated analyses within prespecified subgroups of country, sex, age (18–30 vs. >30 years), baseline viral suppression status and baseline PEth (⩽200 ng/ml vs. PEth>200 ng/ml). The statistical analysis plan was fully pre‐specified (Text S1). The supporting CONSORT checklist is available as supplemental information (Text S2).

### 2.12 Ethical approval

This trial was approved by the ethical review boards of Makerere University (Uganda), Kenya Medical Research Institute (Kenya) and the University of California San Francisco (USA). All participants provided written informed consent at enrolment.

## 3 RESULTS

### 3.1 Enrolment

**Figure 1:**
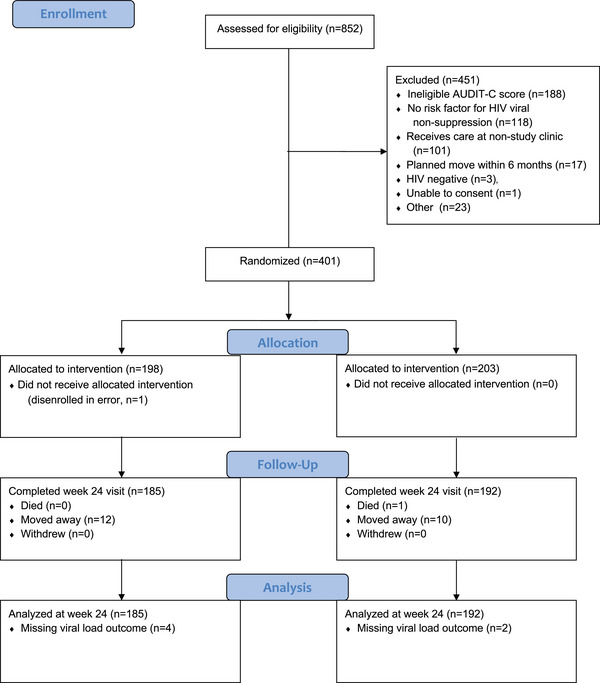
CONSORT diagram. Abbreviation: AUDIT‐C, Alcohol Use Disorders Identification Test—Consumption.

We screened 852 adults; 401 (47%) met eligibility criteria, were enrolled and randomized: 203 control, 198 intervention (Figure 1). One intervention participant was erroneously dis‐enrolled at baseline and excluded from the analyses.

Among study participants, 131 (33%) were female, median age was 37 (interquartile range [IQR] 31–43) years and 286 (71%) had completed primary education or less (Table 1). Sixty (15%) participants were recruited from alcohol‐serving venues in Kenya, 110 (27%) from HIV clinics in Kenya and 230 (58%) from HIV clinics in Uganda. Participants met eligibility criteria for risk of HIV viral non‐suppression for recent unsuppressed viral load (*n* = 141, 35%), missed clinic visit(s) (*n* = 111, 28%), new HIV diagnosis (*n* = 109, 27%) and re‐engaging in care (*n* = 39, 10%). At baseline, 321 (80%) participants were on ART, including those with a new HIV diagnosis. Among those with a prior HIV diagnosis, 279 (96%) were on ART. Overall, 239 (60%) participants were virally suppressed (HIV viral load ≤400 copies/ml) at baseline.

**Table 1:** Baseline characteristics

	Intervention	Control	Total
	*N* = 197	*N* = 203	*N* = 400
**Female**, *n* (%)	60 (30)	71 (35)	131 (33)
**Age**, median [Q1,Q3]	37 [31,45]	37 [30,42]	37 [31,43]
**Age 18–30**, *n* (%)	42 (21)	52 (26)	94 (24)
**Country**, *n* (%)			
Kenya	84 (43)	86 (42)	170 (42)
Uganda	113 (57)	117 (58)	230 (57)
**Marital status**, *n* (%)			
Married	116 (59)	124 (61)	240 (60)
Widowed/divorced/separated	60 (30)	59 (29)	119 (30)
Never married	21 (11)	19 (9)	40 (10)
**Education**, *n* (%)			
Less than primary	8 (4)	9 (4)	17 (4)
Primary	131 (66)	138 (68)	269 (67)
Secondary	47 (24)	46 (23)	93 (23)
Post‐secondary	11 (6)	10 (5)	21 (5)
**Literacy level**, *n* (%)			
Cannot read at all	28 (14)	29 (14)	57 (14)
Can read parts of a sentence	38 (19)	40 (20)	78 (20)
Can read whole sentence	129 (65)	131 (65)	260 (65)
**Alcohol‐related exposures in job**, *n* (%)	18 (9)	26 (13)	44 (11)
**Recruitment site**, *n* (%)			
Alcohol‐serving venue	32 (16)	28 (14)	60 (15)
HIV clinic	165 (84)	175 (86)	340 (85)
**Enrolment criteria**, *n* (%)			
Unsuppressed viral load	70 (36)	71 (35)	141 (35)
Missed visit	52 (26)	59 (29)	111 (28)
New to care	50 (25)	59 (29)	109 (27)
Re‐engaging in care	25 (13)	14 (7)	39 (10)
**On ART with prior HIV diagnosis**, *n* (%)	141 (96%)	138 (96%)	279 (96%)
**On ART at baseline**, *n* (%)	159 (81)	162 (80)	321 (80)
INSTI‐based regimen	113 (71)	115 (71)	228 (71)
NNRTI‐based regimen	6 (4)	8 (5)	14 (4)
PI‐based regimen	22 (14)	18 (11)	40 (12)
On ART, regimen unknown	18 (11)	21 (13)	39 (12)
**Baseline viral load > 400 copies/ml**, *n* (%)	79 (40)	82 (40)	161 (40)
**Baseline AUDIT‐C score**, median [Q1,Q3]	6 [4,8]	6 [4,8]	6 [4,8]
**Baseline PEth (ng/ml)**, mean [95% CI]	492 [398–585]	481 [396–566]	486 [423–550]
**Baseline PEth**, *n* (%)			
PEth ⩽ 200 ng/ml	90 (45)	90 (44)	180 (45)
PEth >200 ng/ml	107 (54)	113 (56)	220 (55)

Abbreviations: ART, antiretroviral therapy; AUDIT‐C, Alcohol Use Disorders Identification Test—Consumption; NNRTI, non‐nucleoside reverse transcriptase inhibitor; NSTI, integrase strand transfer inhibitor; PEth, phosphatidylethanol; PI, protease inhibitor: Q1, quartile 1; Q3, quartile 3.

The median baseline AUDIT‐C score was 6 (IQR 4–8). The mean baseline PEth was 486.7 ng/ml (95% CI: 423.9–549.6), which is above the recommended cutoff for chronic excessive alcohol use (200 ng/ml) [30]. In 283 (71%) participants, PEth was ≥50 ng/ml, consistent with unhealthy drinking [29]. At baseline, 98% (*n* = 391) of participants met the definition for unhealthy alcohol use; a subset of participants (*n* = 9, 2%) reported lower AUDIT‐C scores after enrolment and had baseline PEth <50 ng/ml.

### 3.2 Follow‐up

Over 24 weeks, 22/400 (6%) participants moved out of study communities (12/197 intervention and 10/203 in control); additionally, one control participant died. Endpoint viral loads were obtained on 371/377 (98%) of the remaining participants: 181/185 (98%) intervention and 190/192 (99%) control. Endpoint PEth was obtained in 181/185 (98%) intervention and 188/192 (98%) control participants.

### 3.3 Intervention adherence and fidelity

Among the 197 intervention arm participants, 196 (99%) completed the baseline in‐person counselling session and 184 (93%) completed session 2. Booster calls were completed for 160 (81%), 148 (75%) and 151 (77%) at 3, 6 and 9 weeks, respectively. Median intervention content fidelity and counselling skills scores (*n* = 29 sessions observed) were 93% (IQR 82–94%) and 83% (IQR 79–92%), respectively.

### 3.4 Primary outcome: viral suppression

From 60% at baseline in both arms, viral suppression increased to 83% (95% CI: 78–89%) in the intervention and 82% (95% CI: 78–87%) in the control arm at 24 weeks (Table 2), yielding a non‐statistically significant improvement of 1% (RR = 1.01, 95% CI: 0.93–1.1). The findings were similar across subgroups defined by age, sex, country, baseline alcohol use, baseline non‐suppression risk factor and baseline HIV viral suppression status. There was a stronger intervention effect among those with baseline unsuppressed viral loads, with higher suppression in the intervention (73%, 95% CI: 64–83) versus control arm (64%, 95% CI: 53–75), yielding a relative improvement of 15% (RR = 1.15, 95% CI: 0.93–1.43, not statistically significant). Results were robust to analytic choice.

**Table 2:** Comparison of HIV viral suppression (<400 copies/ml) between arms at 24 weeks, overall and by subgroups

	Intervention, % (95% CI)	Control, % (95% CI)	RR (95% CI)
**Overall**	83 (78−89)	82 (77−87)	1.01 (0.93−1.1)
**Sex**			
Female	93 (86−100)	93 (88−98)	1 (0.91−1.1)
Male	79 (72−86)	78 (71−84)	1.02 (0.91−1.16)
**Age**			
18–30 years old	89 (79−98)	83 (73−92)	1.07 (0.91−1.25)
31 years or older	83 (77−88)	82 (76−87)	1.01 (0.91−1.12)
**Country**			
Uganda	88 (82−94)	82 (75−88)	1.08 (0.97−1.2)
Kenya	78 (68−86)	83 (76−91)	0.93 (0.8−1.07)
**Baseline viral suppression**			
Viral load suppressed (≤400 copies/ml)	91 (85−96)	95 (91−99)	0.96 (0.89−1.03)
Viral load non‐suppressed (>400 copies/ml)	73 (64−83)	64 (53−75)	1.15 (0.93−1.43)
**Baseline Peth**			
PEth ⩽ 200 ng/ml	87 (80−94)	84 (77−91)	1.03 (0.92−1.16)
PEth >200 ng/ml	81 (73−89)	80 (74−87)	1.01 (0.89−1.14)

Abbreviations: CI, confidence interval; PEth, phosphatidylethanol.

### 3.5 Secondary outcomes

We found significant reductions in unhealthy alcohol use (AUDIT‐C positive or PEth≥50 ng/ml) at 24 weeks in the intervention compared to control. Unhealthy alcohol use declined from 98% at baseline in both arms to 73% (95% CI: 68–78%) in the intervention and 84% (95% CI: 80–89%) in the control, yielding a relative reduction of 14% (RR = 0.86, 95% CI: 0.79–0.94; Table 3).

**Table 3:** Comparison of unhealthy alcohol use (drinking above recommended limits) [4] at 24 weeks, overall and by key subgroups

	Intervention, % (95% CI)	Control, % (95% CI)	RR (95% CI)
**Overall**	73 (68−78)	84 (80−89)	0.86 (0.79−0.94)
**Sex**			
Female	53 (42.5−62.5)	75 (66−84)	0.7 (0.56−0.88)
Male	82 (77−88)	89 (84−94)	0.93 (0.85−1.01)
**Age**			
18–30 years old	58 (47−68)	79 (69−90)	0.72 (0.58−0.91)
31 years or older	77 (71−82)	86 (82−91)	0.89 (0.81−0.97)
**Country**			
Uganda	77 (70−83)	89 (84−95)	0.86 (0.77−0.96)
Kenya	68 (60−76)	77 (70−85)	0.87 (0.75−1.02)
**Baseline viral suppression**			
Viral load suppressed (≤400 copies/ml)	74 (67−80)	82 (77−88)	0.89 (0.80−0.99)
Viral load non‐suppressed (>400 copies/ml)	72 (65−80)	88 (82−95)	0.82 (0.72−0.93)
**Baseline Peth**			
PEth ≤200 ng/ml	46 (37−56)	68 (59−77)	0.68 (0.53−0.87)
PEth>200 ng/ml	95 (90−99)	98 (95−100)	0.97 (0.92−1.02)

Abbreviations: CI, confidence interval; PEth, phosphatidylethanol; RR, risk ratio.

Significant reductions in unhealthy alcohol use were seen in most subgroups (Table 3). The effects were stronger among women (RR = 0.70, 95% CI: 0.56–0.88) than men (RR = 0.93, 95% CI: 0.85–1.01) and for persons 18–30 years (RR = 0.72, 95% CI: 0.58–0.91) versus those over 31 years (RR = 0.89, 95% CI: 0.81–0.97). There was a relative reduction in 24‐week unhealthy alcohol use of 32% (RR = 0.68, 95% CI: 0.53–0.87) among participants with a baseline PEth⩽200 ng/ml, but no difference between arms among participants with a baseline PEth >200 ng/ml (RR = 0.97, 95% CI: 0.92–1.02).

## 4 DISCUSSION

In this RCT, a brief culturally adapted alcohol counselling intervention over 12 weeks did not increase the likelihood of viral suppression at 24 weeks among adult PWH with unhealthy alcohol use and a high risk of HIV viraemia. Viral suppression at 24 weeks increased from 60% to over 80% in both intervention and control participants. Unhealthy alcohol use declined in both study arms with a significant 14% relative reduction in the intervention arm compared to the control arm.

There are several potential explanations for our main findings. First, a 14% reduction in unhealthy alcohol use may have been insufficient to drive differences in viral suppression among intervention participants. Alcohol use has been linked to decreased viral suppression among PWH [8, 37], especially at higher use levels [8, 9, 37]. However, it is unknown what degree of reduction in alcohol use is needed to translate to viral suppression changes. Recent studies on behavioural alcohol interventions impacting viral suppression among PWH have shown mixed results. A recent RCT in Uganda of a similar intervention for PWH and unhealthy alcohol use found viral suppression was 93–95% across trial arms at follow‐up, despite a significant reduction in self‐reported number of drinking days in intervention participants [17]. However, baseline viral suppression was >85% and endpoint PEth did not change between arms. In contrast, an RCT of an intervention delivered over 8 weeks to men with HIV in Vietnam showed increases in self‐reported alcohol abstinence and viral suppression at 12 months. The latter was not detected at 3‐ or 6‐month timepoints, potentially suggesting our 24‐week follow‐up was too short to detect long‐term impacts on viral suppression [34]. A 2017 meta‐analysis of alcohol counselling interventions among PWH found small but significant reductions in alcohol consumption and increases in viral suppression [20], though a 2019 systematic review found no effect on either [21].

Second, the lack of effect on viral suppression may be because modern ART regimens containing integrase strand transfer inhibitors (INSTIs) are robust and may yield viral suppression even with suboptimal adherence [38] due to active alcohol use. Furthermore, INSTI‐containing regimens exhibit faster times to viral suppression than other backbone regimens [39], which may result in suppression soon after restarting ART, such as shortly before clinic/study visits. Alcohol use has been shown to lower rates of viral suppression via decreased ART adherence [37]; however, this has not been demonstrated in the era of INSTIs. In our trial, 71% of participants on baseline ART were on an INSTI. While the alcohol reduction demonstrated in intervention participants may have translated to ART adherence improvements, it may not have been enough to produce differences in viral suppression.

Third, factors outside of the alcohol counselling intervention may have driven the high viral suppression in both arms. Our study procedures, which identified unhealthy alcohol use through screening, provided brief alcohol counselling in the control, gave viral load feedback and gave transportation reimbursements, coupled with universal ART eligibility may have been sufficient to increase engagement in care and adherence, yielding the increase in viral suppression from 60% to >82% in both arms. Additionally, adaptations to HIV care provision due to COVID‐19 pandemic restrictions and increased provision of differentiated care models [40], including spaced visits and longer refills, may have improved care engagement and viral suppression for persons with alcohol use [41].

Our trial resulted in a significant reduction in unhealthy alcohol use among intervention participants compared to control. This is one of the first alcohol intervention trials, to our knowledge, to incorporate PEth into the alcohol outcome measure. The composite measure of unhealthy alcohol, comprised of self‐report and an alcohol biomarker (i.e. AUDIT‐C ≥3 for women, ≥4 for men or PEth ≥50 ng/ml), helps to overcome limitations in the sensitivity of either measure alone and to reduce bias introduced by potential discrepancies in self‐report and PEth that have been demonstrated in PWH in observational studies in Uganda [29, 42, 43, 44] and intervention trials in Kenya [45] and Uganda [17]. We found that the reductions in unhealthy alcohol use were most pronounced among participants with lower PEth levels at baseline, consistent with previous studies of brief counselling‐based interventions [32]. Of note, unhealthy alcohol use rates remained high at 24 weeks, suggesting that additional strategies are needed, especially for those with higher levels of use. Potential strategies that may be combined with brief intervention and adapted to low‐resource settings include pharmacologic interventions [46], incentive‐based interventions, such as contingency management [47], and prevention efforts [48].

Our trial has several limitations. In this individual RCT, there was potential for contamination between trial arms if participants discussed or shared counselling resources [49], which may have impacted alcohol reduction or viral suppression among control participants. Secondly, COVID‐19 pandemic‐related government restrictions on movement, commerce and alcohol‐serving venue closures during the trial may have affected alcohol use [50], care engagement and medication adherence [51] in manners that were not measured, biasing results towards the null. Thirdly, the potential impact of reduced alcohol use among intervention participants on the burden of HIV‐ and alcohol‐associated co‐morbidities [52], financial wellbeing [22] and intimate partner violence [22] was not measured and should be considered in future trials. Lastly, control arm participants with baseline viral non‐suppression received viral load counselling based on laboratory testing done for the trial, potentially accelerating adherence interventions compared to usual care and improving viral suppression, affecting the generalizability of our findings. Our trial had two unique strengths. First, using a composite measure of an objective alcohol biomarker and self‐report helped to eliminate the risk of social desirability bias that may occur with self‐report alone and was a novel approach compared to other studies [42, 53, 54]. Second, our heterogeneous study population, including several risk groups for non‐suppression, a range of unhealthy alcohol use and many female participants, allowed for subgroup analyses to better understand our findings and hone future interventions.

## 5 CONCLUSIONS

In summary, a brief, skill‐based alcohol counselling intervention offered over 3 months had no effect compared to the control condition on 24‐week viral suppression among PWH engaged in unhealthy alcohol use, despite reducing levels of unhealthy alcohol use. Our findings highlight that PWH with unhealthy alcohol use in SSA can achieve high levels of viral suppression and, with a brief alcohol intervention, can reduce alcohol use, potentially decreasing associated co‐morbidities and improving health overall.

## COMPETING INTERESTS

The authors report no competing interests.

## AUTHORS’ CONTRIBUTIONS

SBP, GC, MLP, DVH, MK and JAH contributed to the study design, data analysis and interpretation, literature search and writing of the manuscript. LBB contributed to the study design, data analysis and interpretation, and writing of the manuscript. FM, FO, JK, JA, AO, EK, GA, EB, SK, DO, MB, JS and SW‐K contributed to the study design, data interpretation and writing of the manuscript. The authors had full access to all the data in the study and take responsibility for the integrity of the data and the accuracy of the data analysis.

## FUNDING

The study was supported by the U.S. National Institutes of Health NIAID/NHLBI/NIMH U01 AI150510 (DVH, MK and MLP), NIAAA K23 AA029045 (SBP) and NIAAA K24 AA022586 (JAH).

## Supporting information

Supporting Information file 2: SEARCH‐Alcohol_SAP_v1.0. Format: Word document. Pre‐specified statistical analysis plan.
**Text S1**: Pre‐specified SAPPHIRE Alcohol Statistical Analysis Plan

Supporting Information file 1: CONSORT 2010 Checklist_JIAS. Format: Word document. Standardized CONSORT checklist for randomized controlled trials.
**Text S2**: CONSORT 2010 checklist of information to include when reporting a randomized trial.

## Data Availability

The data that support the findings of this study are available from the corresponding author upon reasonable request.
