# Supplementary material for: Effect of a brief alcohol counselling intervention on HIV viral suppression and alcohol use among persons with HIV and unhealthy alcohol use in Uganda and Kenya: a randomized controlled trial
Source: J Int AIDS Soc. 2023 Dec 6;26(12):e26187. doi: 10.1002/jia2.26187 (PMC10698822; doi:10.1002/jia2.26187)
Supplement: Supplementary file 1 — Supporting Information file 2: SEARCH‐Alcohol_SAP_v1.0. Format: Word document. Pre‐specified statistical analysis plan. Text S1: Pre‐specified SAPPHIRE Alcohol Statistical Analysis Plan [file JIA2-26-e26187-s002.docx]

Sustainable East Africa Research in Community Health

(SEARCH) Collaboration

Statistical Analysis Plan for

Dynamic Choice Care Intervention for Mobile Persons with HIV

in Phase A of SEARCH-Sapphire

Laura B. Balzer, PhD^1^

and the SEARCH Study Team^2^

May 19, 2022

v1.0

^1^University of California, Berkeley

^2^www.searchendaids.com

**Table of Contents:**

[1. Study Overview 2](#_Toc129360418)

[2. Population and Characteristics 3](#_Toc129360419)

[3. Endpoint Measurement and Definition 3](#_Toc129360420)

[4. Evaluation of the SEARCH Intervention Effect 4](#_Toc129360421)

[5. Intervention Fidelity and Implementation 5](#_Toc129360422)

[Appendix: Power calculations 5](#_Toc129360423)

[References 6](#_Toc129360424)

# 1. Study Overview

In Phase A of SEARCH-Sapphire (NCT04810650), we are conducting an individually randomized controlled trial to evaluate the effect of an alcohol counseling intervention for persons with HIV (PWH) and unhealthy alcohol use in rural Kenya and Uganda. Details of the trial design and procedures can be found in the corresponding Study Protocol. Analyses plans for qualitative outcomes and cost-effectiveness outcomes are available elsewhere. Power calculations are given in the Appendix.

In brief, from April through September 2021, we 401 enrolled PWH with unhealthy alcohol use and risk of viral non-suppression. These participants were randomized to the intervention or the standard-of-care using a stratified random block design with stratification factors of country and sex and with random block sizes of 2 and 4. The randomization list was generated by an independent researcher.

The intervention is culturally-adapted and skills-based alcohol counseling, consisting of in-person sessions at baseline and every 12 weeks as well as phone-based “booster” calls every 3 weeks between in-person sessions. The intervention is delivered by lay counselors trained by a licensed clinical psychologist, who also provides ongoing supervision and feedback.

**The primary objective is to evaluate if the intervention improved viral suppression (<400 copies/mL) among PWH with unhealthy alcohol use after 24 weeks of follow-up.** Secondary endpoints, compared between randomized arms at 24-weeks, include alcohol use, as measured by Alcohol Use Disorders Identification Test–Consumption [AUDIT-C] or phosphatidylethanol [PEth]. Additionally, within the intervention arm, we will report completion of counseling sessions and their fidelity.

# 2. Population and Characteristics

The population of interest is PWH who are

- Aged 18+ years
- Enrolled or new to HIV care
- At risk of viral non-suppression:
  - HIV RNA>400 copies/mL in the past 12 months
  - Missed visit (>2 weeks and <90 days from scheduled appointment) in the past 6 months
  - Re-engaging in care (>90 days from last scheduled visit) in the past 6 months
  - New HIV diagnosis (not yet on ART or started ART within past 1 month)
- Have unhealthy alcohol use: AUDIT-C over the past 3-months of ≥3 for women or ≥4 for men

To characterize measurement of this population, we will provide a participant flow diagram (i.e., a CONSORT diagram). Overall and stratified by trial arm and further by sex, we will summarize the baseline characteristics, including sex, age, country, marital status, occupation, education level, literacy level, trial recruitment site (i.e., venue-based or clinic), trial enrollment criteria, baseline ART status and regimen, baseline viral suppression status, and baseline alcohol use via AUDIT-C score and PEth levels. We will categorize age as “younger” if aged 18-30 years. When treated continuously, PEth will be log-10 transformed and values below the limit of quantification (8 ng/mL) set to 0.

# 3. Endpoint Measurement and Definition

The **primary endpoint** is HIV viral suppression (HIV RNA<400 copies/mL) at 24-weeks. The primary analytic population will consist of all study participants residing in the study region. In other words, the primary analysis will exclude persons who died, withdrew, or moved out of the study region. In the primary analysis, missing endpoint viral loads will be treated as failures (i.e., unsuppressed). In pre-specified sensitivity analyses, described below, we will assess the robustness of these analytic choices.

We will also examine the following **secondary endpoints** to capture alcohol use at 24-weeks:

- Composite measure of unhealthy alcohol use: AUDIT-C ≥3 for women and ≥4 for men or PEth ≥50 ng/mL
- AUDIT-C ≥3 for women and ≥4 for men
- PEth ≥ 50ng/mL
- Composite measure of unhealthy alcohol use: AUDIT-C ≥6 or PEth >200 ng/mL
- AUDIT-C ≥6
- PEth >200 ng/mL
- Log-10 transformed PEth (treated continuously)

Pending data availability, we will define the primary and secondary endpoints analogously at 48-weeks.

# 4. Evaluation of the SEARCH Intervention Effect

We will assess the intervention effect with targeted minimum loss-based estimation (TMLE), which improves precision and power by adaptively adjusting for baseline outcome predictors.^1–5^ Here, we will use **TMLE with Adaptive Pre-specification** to flexibly control for baseline covariates, while maintaining Type-I error control and accounting for the randomization scheme.^6–8^ Using 10-fold cross-validation, we will chose the optimal approach for estimating the outcome regression (i.e., the expected outcome given the randomization arm and adjustment covariates) and the known propensity score (i.e., the conditional probability of being randomized to the intervention given the adjustment covariates). Specifically, we will select the combination of estimators (adjustment variables + approach) that minimizes the cross-validated variance estimate.

Our pre-specified, candidate adjustment variables consist of sex, age, country, baseline viral suppression status, baseline PEth (log-10 transformed), baseline AUDIT-C score, and nothing (i.e., unadjusted). Our pre-specified, candidate learners consist of generalized linear models (GLMs) adjusting for a single variable beyond the intervention indicator, stepwise regression, multivariate adaptive regression splines (MARS), MARS after screening based on outcome correlations, and the arm-specific mean outcome.

Primary effect estimates will be for the study sample and on the **ratio scale**: $\frac{1}{n}\sum_{i=1}^{n} Y_{i}\left( 1 \right)\div\frac{1}{n}\sum_{i=1}^{n} Y_{i}\left( 0 \right)$, where $Y_{i}\left( 1 \right)$ denotes the counterfactual outcome for participant *i* under the intervention and $Y_{i}\left( 0 \right)$ denotes the counterfactual outcome for participant *i* under the control*.*^9–11^ Secondary comparisons will be on the difference scale.

We will test the **null hypothesis** that the intervention did not improve viral suppression at 24-weeks using a one-sided test at the 5% significance level. We will also report point estimates and 95% confidence intervals for each effect measure and the arm-specific outcomes at baseline and 24-weeks. Standard error estimation will be based on the estimated influence curve, and statistical inference will follow from the Central Limit Theorem (i.e., using the standard normal distribution).^1^

**Secondary analyses:** To assess the robustness of these findings, we will repeat these analyses using the unadjusted effect estimator. We will also repeat these analyses including persons who moved out of the study region, excluding persons with missing endpoints, and using TMLE to adjust for missing endpoints.

**Subgroup analyses:** We will repeat these analyses within strata defined by country, sex, age group, trial recruitment site, trial enrollment criteria, baseline viral suppression status, and baseline alcohol use: AUDIT-C score 3-5/women or 4-5/men, AUDIT-C score 6-7, AUDIT-C score 8+, PEth<8 ng/mL (undetectable), PEth$\geq$8 ng/mL, PEth<50 ng/mL, PEth$\geq$50 ng/mL, PEth$\leq$200 ng/mL, Peth>200 ng/mL. In subgroups analyses, we will limit the candidate estimation approaches to main terms adjustment for a single covariate or the simple mean and use leave-one-out cross-validation for subgroups with <40 participants. To further understand effect heterogeneity, we may conduct variable importance measures (unadjusted and adjusted) to understand baseline predictors of endline viremia, overall and by arm.

**Secondary endpoints compared by arm:** We will implement analogous analyses to evaluate the intervention effect on alcohol use. In these analyses, the primary approach will exclude persons with missing measures of alcohol use at 24-weeks. We will again examine the robustness of this approach (i.e., adjust with TMLE for missing measures and include persons who moved out of the study region). When examining the effect on log-10 PEth values, we will use the difference scale.

# 5. Intervention Fidelity and Implementation

Within the intervention arm, we will evaluations of content fidelity and counseling skills at randomly selected in-person sessions. We will also report intervention adherence, as the proportion of participants who completed each counseling session.

# Appendix: Power calculations

Sample size and power calculations were based on a two-sample test of proportions with *power.prop.test* function in *R*.^12^ We expect these calculations to be conservative, because of the precision gained through stratified randomization, covariate adjustment during the analysis, and our pre-specified use of a one-sided hypothesis test.

We estimated 200 participants/arm would provide 80% power to detect at least a 14% absolute increase in viral suppression at 24-weeks from 40% under the standard-of-care. As shown in the following Figure, even with 25% attrition (from 200 to 150 participants/arm) and lower or higher than suppression in the control, these calculations suggest we would be well-powered to detect at least a 16% absolute increase in viral suppress.


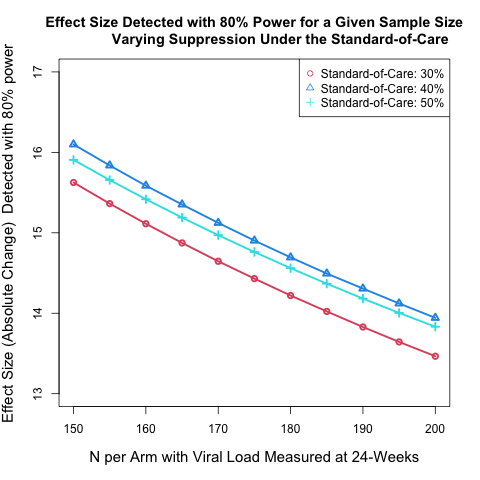


# References

1. van der Laan M, Rose S. *Targeted Learning: Causal Inference for Observational and Experimental Data*. Springer; 2011.

2. Moore KL, van der Laan MJ. Covariate Adjustment in Randomized Trials with Binary Outcomes: Targeted Maximum Likelihood Estimation. *Stat Med*. 2009;28(1):39-64. doi:10.1002/sim.3445

3. Rosenblum M, van der Laan MJ. Simple, Efficient Estimators of Treatment Effects in Randomized Trials Using Generalized Linear Models to Leverage Baseline Variables. *Int J Biostat*. 2010;6(1):Article 13. doi:10.2202/1557-4679.1138

4. van der Laan MJ, Rose S. *Targeted Learning in Data Science*. Springer; 2018.

5. Benkeser D, Díaz I, Luedtke A, Segal J, Scharfstein D, Rosenblum M. Improving precision and power in randomized trials for COVID-19 treatments using covariate adjustment, for binary, ordinal, and time-to-event outcomes. *Biometrics*. 2021;n/a(n/a):1-15. doi:10.1111/biom.13377

6. Balzer L, van der Laan MJ, Petersen M, SEARCH Collaboration. Adaptive Pre-specification in Randomized Trials With and Without Pair-Matching. *Stat Med*. 2016;35(10):4528-4545. doi:10.1002/sim.7023

7. Havlir DV, Balzer LB, Charlebois ED, et al. HIV Testing and Treatment with the Use of a Community Health Approach in Rural Africa. *N Engl J Med*. 2019;381(3):219-229.

8. Balzer LB, van der Laan M, Ayieko J, et al. Two-Stage TMLE to Reduce Bias and Improve Efficiency in Cluster Randomized Trials. *Biostatistics*. 2021;kxab043. https://doi.org/10.1093/biostatistics/kxab043

9. Neyman J. Sur les applications de la theorie des probabilites aux experiences agricoles: Essai des principes (In Polish). English translation by D.M. Dabrowska and T.P. Speed (1990). *Stat Sci*. 1923;5:465-480.

10. Rubin DB. Comment: Neyman (1923) and Causal Inference in Experiments and Observational Studies. *Stat Sci*. 1990;5(4):472-480.

11. Balzer LB, Petersen ML, van der Laan MJ. Targeted estimation and inference of the sample average treatment effect in trials with and without pair-matching. *Stat Med*. 2016;35(21):3717-3732. doi:10.1002/sim.6965

12. R Core Team. *R: A Language and Environment for Statistical Computing*. R Foundation for Statistical Computing; 2022. http://www.R-project.org
